# Unilateral hearing loss: CROS fitting

**DOI:** 10.5935/1808-8694.20130093

**Published:** 2015-10-08

**Authors:** Maria Fernanda Capoani Garcia Mondelli, Lia Auer Hoshii, Tatiana Manfrini Garcia, Regina Tangerino de Souza Jacob

**Affiliations:** a PhD (Professor, Dentistry School of Bauru, University of São Paulo - USP).; b MS. (Product Manager - Phonak Hearing Aids - Brazil).; c MSc Student in Speech and Hearing Therapy - Dentistry School of Bauru - USP (Speech and Hearing Therapist - Speech and Hearing Therapy Clinic - Dentistry School of Bauru - USP). Bauru School of Dentistry - University of São Paulo (USP).

**Keywords:** hearing aids, hearing loss, unilateral, questionnaires

## INTRODUCTION

Unilateral hearing loss is characterized by difficulties in speech perception in noise, difficulty in sound localization and increased overall effort to understand speech.

Studies performed on the deficits of patients with unilateral hearing loss, reported more difficulties when the sound or speech origin came from the impaired side, presumably due to a reduced exploration of binaural information processing^1^.

CROS (Contralateral Routing Signal) device fitting is considered an option for patients with this type of loss. A microphone is placed in the impaired ear and sends a signal via wireless technology to a receiver placed in the normal hearing ear^2^.

The aim of this study is to assess the performance of a patient with the CROS amplification system.

## CASE PRESENTATION

T.C.B.M. developed mumps at 5 years of age. Audiological tests indicated profound right-side sensorineural hearing loss and normal hearing on the left.

In 2011, T. then was 19 years old, a college student in a speech and hearing therapy program, sought assistance complaining of a difficulty to understand the professor in the classroom, communication difficulties in noisy environments and impaired sound localization. She was referred to CROS SSmart Audéo IX fitting.

To assess the benefits and satisfaction with the device, the following procedures were performed:

1. HHIA (“Hearing Handicap Inventory for Adults”) questionnaire before and 3 months after the fitting;
2. Sound localization questionnaire;
3. Measurements using a probe microphone;
4. Evaluation of speech perception in noise.

The HHIA^3^ consists of 25 items, 13 of which involve emotional aspects and 12 social and situational ones. A high score suggests a significant perception of the hearing handicap by the subject.

The Sound localization questionnaire^4^ is used with and without the ISAD, it consists of 14 questions related daily life activities and four possible answers with values ranging between 1 and 4. The value 4 (four) is indicative of a lower degree of difficulty.

In using the probe microphone for the measurements, there should be two reference microphones - paramount to accurately compare the amplification device output when the sound is presented to the better ear versus the impaired ear. The probe microphone is inserted only in the normal hearing ear - the one that has an output from the CROS system.

For the HINT^5^, assessment, T. should be able to recognize and repeat simple sentences in silence and in noise. The follow situations were assessed: speech and noise in front: 0°; frontal speech and left-side noise 90°; frontal speech and right-side noise: 90°.

## DISCUSSION

The patient had results consistent with a significant handicap in the HHIA questionnaire (46%); with a greater hearing difficulty in social situations, which is common for such environments to be noisy.

After using the ISAD for 3 months for about 10 to 12 hours per day, the HHIA results were considered absent (8%), indicating a benefit of fitting a CROS.

Sound localization is affected because individuals with unilateral hearing loss do not have the benefit of interaural time: when a sound comes from one direction, the interaural time difference and phase differences of continuous sounds in both ears allows the individual to determine which direction the sound is coming from^6^.

In the questionnaire, T. showed a value indicating lower degree of difficulty with the use of hearing aids (3.32) vis-à-vis the absence of amplification (1.67). Fitting a CROS should be indicated for patients who are able to control the location and positioning of the head in relation to an undesirable noise, optimizing device use^3^.

The probe microphone measurements allowed to objectively check the CROS operating system and the elimination of the head shadow effect.

**Chart 1:**
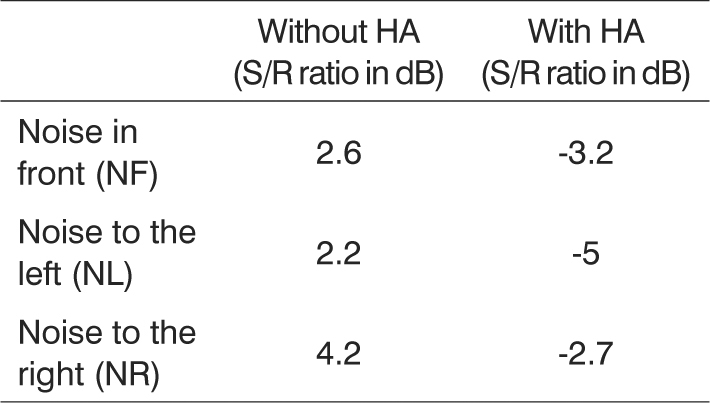
HINT results with and without hearing aids.

The HINT test results before and after fitting are depicted on Chart 1.

## FINAL REMARKS

The profound unilateral sensorineural hearing loss proved to impair the daily life of the patient, and with the CROS fitting there were improvements related to the handicap, sound localization ability, head shadow effect and in speech discrimination.
